# Stay calm in crowds: Avoiding emotional faces in ensemble perception

**DOI:** 10.3758/s13414-025-03124-4

**Published:** 2025-07-10

**Authors:** Xin Luo, Megan Bartlett, Michael E. R. Nicholls

**Affiliations:** 1 College of Education, Psychology and Social Work, Flinders University, Sturt Road, Bedford Park, SA 5042 Australia; 2 School of Psychology, Southwest University, Chongqing, China; 3 School of Psychology, The University of Adelaide, Adelaide, Australia

**Keywords:** Facial expressions, Ensemble perception, Attentional biases, Emotional processing

## Abstract

Previous research has shown that people tend to display attentional biases toward faces with strong emotions within crowds, often overestimating the extremity of the average emotional expression. However, this emotional amplification effect has not been consistently observed in tasks where observers summarize other crowd features, such as the number of faces. This study aims to explore the attentional mechanisms underlying these inconsistent findings. To do so, we recruited 584 participants across four online experiments and employed an equivalence judgment task to assess participants’ ability to estimate the number of emotional faces. In the task, participants determined whether the number of two types of facial expressions within a series of crowds was the “same” or “different.” Experiment 1 revealed that the number of emotional faces (angry and happy) was underestimated relative to neutral faces. Experiment 2 replicated this underestimation effect across different face types and exposure durations. Experiment 3 demonstrated that the emotional amplification effect may be caused by strong emotion contrasts within crowds. Experiment 4 confirmed that the underestimation of the number of emotional faces could be replicated in the numerosity estimation task with different instructions. Our findings suggest that people may strategically suppress attention to emotional faces to mitigate their emotional response. This study provides important empirical evidence to enhance our understanding of the cognitive processes underlying emotion perception and social behavior.

The perception of facial expressions plays an important role in daily life, as it drives people’s behavior during social interactions. Many social activities take place in group settings, such as finding a friendly person to talk to at a cocktail party or assessing engagement during public speaking based on facial expressions. Over the past decades, many studies using the face-in-the-crowd task have demonstrated that a single emotional face is detected more efficiently than a neutral face in a crowd (Savage et al., 2013). However, the role of attentional biases to emotional faces in the processing of multiple emotional expressions within a crowd remains unclear.

The face-in-the-crowd task is a widely applied method for measuring the efficiency of detecting emotional faces. Hansen and Hansen (1988) first introduced this task, asking participants to detect whether a discrepant face was present among a crowd of other facial expressions. Their findings revealed that angry faces were detected faster than happy faces, suggesting an attentional bias toward angry faces. This advantage in the detection of angry faces is referred to as “the angry superiority effect” and has been supported by several studies (Eastwood et al., 2001; Hahn et al., 2006; Horstmann & Bauland, 2006; Öhman et al., 2001). However, other researchers have reported a happy superiority effect and argued that the angry superiority effect was caused by visual feature confounds (Becker et al., 2011; Savage et al., 2013). For example, Hansen and Hansen (1988) inadvertently introduced a black blotch at the base of each angry face when converting photographs into pixelated images. After this artifact was removed, Purcell et al. (1996) found a “happy superiority effect,” with happy faces detected more rapidly than angry faces. Moreover, the happy superiority effect was more commonly observed when using photographic faces (Becker et al., 2011; Savage et al., 2013), while the angry superiority effect seems to be primarily limited to studies using schematic faces (Savage et al., 2013). These mixed results show that subtle low-level features can affect the search process (Nummenmaa & Calvo, 2015).

While the face-in-the-crowd task provides valuable insights into the research on attentional biases toward emotional faces, this area still needs further exploration. Beyond the potential confounding effects of visual features, researchers have argued that the crowd effect may confound the target effect, leading to ambiguous results from the face-in-the-crowd task (Bucher & Voss, 2019). Hahn et al. (2006) presented angry targets among either happy or neutral distractor faces and found that search performance for an angry face among neutral distractor faces was faster than among happy distractor faces. Additionally, studies have shown that negative crowds are searched more slowly than positive crowds on trials where no target is present (Hansen & Hansen, 1988; Horstmann & Bauland, 2006). The evidence suggests that the evaluation of the distractor background may influence detection efficiency by capturing attention and delaying disengagement (Bucher & Voss, 2019; Frischen et al., 2008). Thus, it is difficult to interpret whether the observed detection advantage for specific emotional expressions found in research is due to the rapid direction of attention to the target or quick evaluation of the distractors. Furthermore, the face-in-the-crowd task does not assess attentional processing when individuals engage with multiple emotional target faces simultaneously. In real-world social scenarios, we often need to process various emotional cues at once. Tasks based on ensemble perception provide an alternative approach for assessing attentional biases to emotional faces in complex and emotionally diverse contexts.

## Ensemble perception

Ensemble perception is the ability to extract an overall statistical representation from a set of similar objects (Ariely, 2001; Whitney & Yamanashi Leib, 2018). Research on ensemble perception has proven that people can quickly and accurately extract representations from various levels of sets, including judging average speed (Watamaniuk & Duchon, 1992), and perceiving average size (Ariely, 2001; Chong & Treisman, 2003). Haberman and Whitney (2007) first demonstrated that people can accurately extract the mean emotional tone from a crowd of faces. In their study, participants were presented with arrays containing four or 16 faces for 2 s and were then asked to judge whether the subsequently presented single test face was happier or sadder than the mean emotion of the previous array. Since all members of the group provide relevant information for extracting features, research on ensemble perception avoids the potential interaction effects between targets and distractors (Bucher & Voss, 2019). Moreover, unlike the face-in-the-crowd task, which is vulnerable to low-level visual features, ensemble perception is a robust phenomenon (Whitney & Yamanashi Leib, 2018): Experiments using different types of face stimuli, including human photos and computer-generated faces, have found consistent outcomes (Bucher & Voss, 2019). However, few studies have applied ensemble perception tasks to examine attentional biases toward emotional faces, and the findings remain inconclusive.

Research has demonstrated that over-attending to emotional faces can lead to a biased statistical representation. Goldenberg et al. (2021) presented face arrays with varying intensities of happy or angry faces and asked participants to estimate the average emotion. They found that the estimation of the average emotion was more extreme than the array actually was (Goldenberg et al., 2021). This result was in line with a previous study, which showed that people overestimate the average emotion when its variance is higher rather than lower (Goldenberg et al., 2020). Goldenberg et al. (2021) attributed these biased representations to people preferentially attending to and sampling the emotional faces within a group. The overestimation of emotional faces, however, has been inconsistently reported in other studies. Bucher and Voss (2019) presented arrays containing angry or happy faces mixed with neutral faces and asked participants to determine whether there were more emotional or neutral faces. They found no evidence of overestimation for both happy and angry faces in general, but participants tended to overestimate angry faces when there were fewer angry faces than neutral faces in the array. As the number of angry faces increased, the overestimation effect disappeared (Bucher & Voss, 2019). Ueda (2022) used a similar task to study this effect. To assess the response biases, they introduced the point of subjective equality (PSE), which refers to the point at which participants perceive two stimuli as equal. They expected to observe that when emotional and neutral faces appeared in equal numbers, participants would more frequently report having more emotional faces within the array. This would show an overestimation of emotional faces. However, the PSE results did not support an overestimation of emotional faces relative to neutral faces. These findings are interesting because Goldenberg et al. (2022) suggested that the amplification effect is robust in arrays with more emotional faces.

## Top-down modulation in emotional perception

Inconsistent findings in ensemble perception research may stem from variations in task instructions, which could influence the processing of emotions. Previous research suggests that top-down modulation in emotion perception can lead to varied attentional responses depending on the emotional context (Sussman et al., 2016). While top-down guidance is commonly thought to facilitate the identification of emotional stimuli (Sussman et al., 2016), some studies suggest that specific emotion instructions can inhibit automatic selective attention toward emotional faces. For example, M. Williams et al. (2005) found that participants detected fear faces slower when asked to search for an emotional face in a crowd, compared with when they were asked to indicate whether the target face was present or absent. Additionally, Lange et al. (2008) asked participants to judge the friendliness of crowds and found participants tended to underestimate the friendliness of crowds containing angry and neutral faces and overestimate the friendliness of crowds containing angry and happy faces. These findings were attributed to prestimulus expectations or goals that prompted participants to strategically allocate attention to both targets and distractors to improve accuracy (Lange et al., 2011; M. Williams et al., 2005). Existing evidence suggests that top-down processes modulate task-relevant attentional allocation, diminishing the bottom-up capture of attention by emotional stimuli and, in turn, reducing bias toward emotional faces.

It is unclear how tasks without specific emotion instructions influence the extraction of statistical features from a crowd. Asking participants to extract the mean or overall emotion is the most common nonspecific emotional task in ensemble perception research. Goldenberg and colleagues consistently found an overestimation of emotional faces in studies where participants were asked to report the average emotion of a crowd (Goldenberg et al., 2021, 2022). In these studies, face stimuli were randomly selected from a set of 50 faces on a continuum of emotional intensity, ranging from neutral (1 point) to extremely angry or happy (50 points). As a result, the crowds presented contained few or no neutral faces. The imbalance of emotional valence may increase attention toward emotional faces, thereby amplifying the emotional effect. Tasks where emotional valence is balanced, however, have rarely observed the emotional amplification effect (Bucher & Voss, 2019; Ueda, 2022; Yang & Baek, 2022; Yang et al., 2013). In these studies, the number of two types of emotional faces in the crowd were systematically varied across several ratios. For example, 12-face crowds comprising angry and neutral faces included three ratios: four, six, and eight angry faces. Instead of extracting the average emotion, these studies explicitly instructed participants to focus on specific emotions, such as identifying whether there were more angry or neutral faces in the crowd. Given the high correlation between mean emotion and number of faces, it is reasonable to assume that if observers perceive the average emotion as more intense than it is, they may judge that there are more emotional faces. Such findings, however, have not been consistently observed in studies that estimate the number of emotional faces. This absence may be attributable to task instructions influencing emotional processing, leading to reduced attentional biases to emotional faces. Using a nonspecific emotion task to test the ability of numerosity estimation of faces may eliminate top-down modulations, revealing an overestimation of the number of emotional faces.

## The current study

In the current study, we aimed to investigate attentional biases in ensemble perception using a task with non-emotion-specific instructions. To do so, we introduced an equivalence judgment task, where participants viewed arrays of neutral and emotional faces and were asked to judge whether the number of two types of emotional faces in the arrays was the same or different. This instruction did not specify any particular emotion, and as the same or different judgment is unrelated to the dimension of interest (more or less) of the emotional faces, it reduces the likelihood of participants guessing the purpose of the study. The arrays were presented with proportions of the two emotional faces increasing (or decreasing) in units of one. To achieve an accurate estimate, participants need to identify a precise distribution (i.e., the proportion of emotional faces) in the array. According to Weber’s law, discrimination of differences in continuous properties (such as brightness, size, time, quantity, etc.) depends on the ratio between the competing stimuli (Stevens, 1957). As the ratio between two types of faces approaches 1:1, participants are more likely to respond “same.” On the contrary, as the ratio decreases, it becomes easier to distinguish the number of faces, participants are more likely to respond “different.” The frequency of "same” responses follows a Gaussian distribution, with the midpoint centered on the actual value where the two types of faces are equal. The PSE was introduced to examine the response bias. Just noticeable differences (JND), the smallest detectable difference between two stimuli, was introduced to examine the precision of judgments.

The main goal of this study was to test whether participants would overestimate the number of emotional faces. We hypothesized that when emotional faces were fewer than neutral faces in an array, participants would more frequently judge the two types of faces as equal in number compared with when emotional faces were more numerous. As a result, the PSE was expected to be lower than the actual value of equal numbers between the two types of faces (e.g., if an array contained 30 faces, the PSE would be less than 15). This hypothesis assumed that in no-emotion-specific tasks, attention is automatically and preferentially directed to emotional faces, potentially leading to more emotional faces than neutral ones being sampled for numerical estimation. Furthermore, because mean, number, and area are closely correlated, participants may infer the number of faces based on the overall emotional tone. Due to the amplification effect of emotional faces, participants may overestimate the average emotion, resulting in a tendency to judge the number of two types of faces as the same when emotional faces are fewer than neutral faces. We also expected that the equivalence judgment task would effectively test the angry or happy superiority effect, providing valuable empirical evidence in this area.

To the best of our knowledge, this is the first study to employ an equivalence judgment task to assess numerosity estimation of emotional faces in ensemble perception and examine the factors influencing these judgments. We calculated the PSE to represent perceptual bias and the JND to indicate precision. This study consists of four experiments. In Experiment 1, we used photographic faces to create face arrays. In Experiment 2, we used schematic faces to control for emotional intensity and manipulated exposure duration. In Experiment 3, we examined how participants perceived happy and angry faces when presented together as competing emotional stimuli. Finally, in Experiment 4, we assessed how different quantity judgment tasks influenced participants' estimations. This study received ethical approval from the human ethics review panel of Flinders University: 4645.

## Experiment 1

The first aim of Experiment 1 was to test whether participants would overestimate the number of emotional faces in an array. Arrays were composed of 30 images of human actors displaying various facial expressions. The reason for presenting arrays of 30 faces was to allow for the optimal ratio of incremental changes between the two types of faces and provide sufficient data for accurately measuring the PSE and the JND. Research has indicated that at least 7–9 ratios spanning both below and above the reference are needed to reach a reliable measurement (Stevens, 1957). In the present study, the actual equal point of the two emotions in the 30-face arrays is 15. An overestimation of the number of emotional faces would be reflected by a PSE shift towards values smaller than 15. The JND served to assess whether presenting different emotional faces could affect the difficulty of this task. A smaller JND would indicate that participants could more precisely judge that the arrays contain a particular emotional face compared with another.

A secondary aim of this experiment was to explore whether mood states impact attentional biases in ensemble perception. The interaction of emotional states and emotional stimuli plays an important role in daily human behavior and functioning. According to Beck’s cognitive model, the cause and maintenance of emotional disorders involves biased processing of emotion-congruent information (Beck & Clark, 1988). This bias manifests as a preference for processing threat-related stimuli in anxiety, while depression is associated with biased processing of negative information such as personal loss and failure (Beck & Clark, 1988; J. M. G. Williams et al., 1988). Furthermore, research suggests that excessive generalization may underlie anxiety disorders (Schechtman et al., 2010), leading to inaccurate perception and discrimination in anxious individuals. El-Bar et al. (2017) conducted a study on perceptual and discriminative stimuli in adolescents with high levels of anxiety, and found that the discrimination ability of the adolescents decreased, which was related to broad generalization of emotional valence. For example, individuals with anxiety would tend to judge a neutral or happy face as threatening. Accordingly, we expected that participants with high levels of anxiety or depression would overattend to the emotional faces, resulting in an overestimation of their number and lower precision than those with low levels of anxiety or depression.

### Method

#### Participants

A total of 200 participants who were fluent in English and had normal or corrected to normal visual acuity were recruited from Prolific (https://www.prolific.co/) and randomly assigned to either the angry or happy condition. Prior to participation, they were provided with a consent form and information sheet through an online tick box. Participants were reimbursed at a rate of US $9.50 per hour for their participation in an experimental session that lasted no longer than 45 min.

The sample size was determined by referring to the number of participants in Yang et al. (2013), as their experimental task and main analysis methods are most comparable with those used in our study. Since the main hypotheses for Experiments 1 and 2 require similar statistical tests, we recruited 100 participants for each condition across these experiments. Due to data exclusion based on predefined criteria, there were slight differences in the actual sample size included in the analysis. Data were excluded from participants who failed to complete the experimental session, had incomplete or missing data, responded “same” or different” for each item on the task, or whose responses were greater than three standard deviations from the mean. The final sample included 98 participants in the angry condition (45 women, *M*_age_ = 26.42 years, *SD* = 7.13) and 93 participants in the happy condition (56 women, *M*_age_ = 29.30 years, *SD* = 7.66).

#### Stimuli

To create the arrays, color photos of 20 male and 20 female actors displaying three different emotional facial expressions (happy, neutral, and angry) were selected from the Karolinska Directed Emotional Faces (KDEF) database (Lundqvist et al., 1998). The experimental task was programed using the Builder interface of PsychoPy (Peirce et al., 2019) and hosted online via Pavlovia (https://pavlovia.org/).

**Fig. 1:**
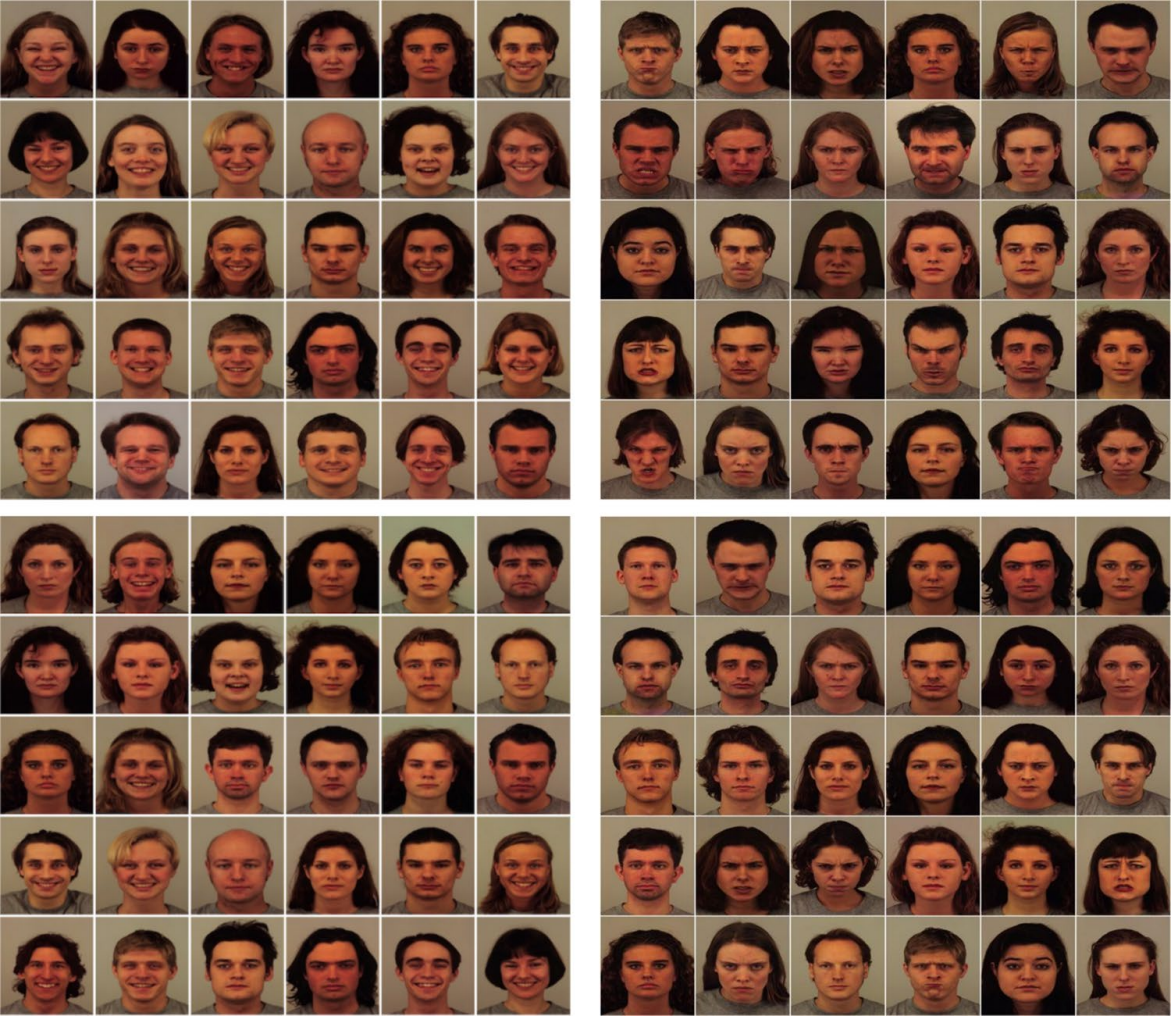
Sample arrays for Experiment 1. *Note.* Happy-dominant (i.e., 20H:10N, top-left panel), angry-dominant (i.e., 20A:10N, top-right panel), and neutral-dominant (i.e., 10H:20N, bottom-left panel; 10A:20N, bottom-right panel) arrays

Each facial image was 130 × 130 pixels in size and randomly arranged in a 6 × 5 grid within each 30-item array. A total of 11 different types of arrays were created, systematically varying the ratio of emotional to neutral faces in increments of one. At the lowest ratio, arrays contained 10 emotional faces and 20 neutral faces, and at the highest ratio, arrays contained 20 emotional faces and 10 neutral faces. Additionally, 20 different arrangements were created for each array ratio, in which the position of the emotional and neutral facial images was randomized. No actor displayed two different facial expressions within one array. The proportion of females and males in the array was balanced (see Fig. 1). In total, there were 220 unique array stimuli (11 array ratios × 20 arrangements) for each emotional condition.

#### Procedure

Participants were asked to complete the experiment on a computer or laptop rather than on a mobile device, such as a phone or tablet. At the beginning of the experiment, participants read a description of the experimental procedure. Instead of completing a block of practice trials, participants were given three example images of arrays comprised predominately of emotional faces, neutral faces, or an equal number of emotional faces, respectively. In each trial, a fixation cross was presented for 1 s, followed by an array for 5 s. Participants were asked to press the “S” or “D” key on the keyboard to indicate whether the number of emotional and neutral faces in the array was the same (S) or different (D). There was no time limit for participants to respond. In the angry condition, participants viewed arrays composed of angry and neutral faces, whereas in the happy condition, participants viewed arrays composed of happy and neutral faces. Participants completed a total of 220 trials, divided into two blocks, with each block consisting of 110 trials.

At the conclusion of the experimental task, participants completed the 21-item version of the Depression, Anxiety and Stress Scale (DASS-21; Lovibond & Lovibond, 1995) to assess their mood state. The DASS-21 includes three subscales: Depression, Anxiety, and Stress, each consisting of seven items. Cronbach’s alphas for the subscales of the DASS-21 were .94 for Depression, .87 for Anxiety, and .91 for Stress (Lovibond & Lovibond, 1995).

## Results and discussion

**Fig. 2:**
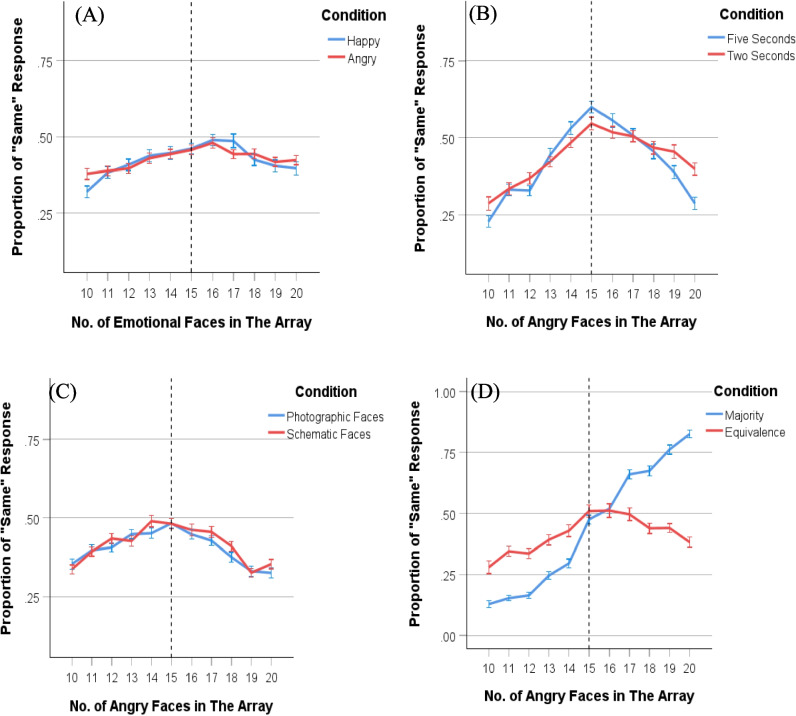
Distribution of “same” responses for each number of emotional faces in the array.** Note.** The distribution of “same” responses for the number of emotional faces in the array from Experiment 1 **(A)**, Experiment 2 **(B)**, Experiment 3 **(C)**, and Experiment 4 **(D)**. Error bars indicate one standard error. (Color figure online)

Analyses were conducted based on the number of “same” responses at each ratio of emotional faces in the array. The response frequency fits a normal distribution curve centered at a value of 15, representing equal numbers of the two types of faces. A rightward skew of the curve indicates a tendency for underestimating the number of emotional faces, while a leftward skew indicates a tendency for overestimating the number of emotional faces (see Fig. 2A for Experiment 1).

To determine judgment biases, an averaging model was applied to calculate the PSE (Bock & Jones, 1968; Guilford, 1954), in which the psychometric formula is defined as follows:

1 $$PSE=\frac{\sum_{i=1}^{k}{{r}_{i}a}_{i}}{{\sum_{i=1}^{k}r}_{i}},$$

where “*r*”represents the participant’s response when there are “*a*” number of emotional faces in the array, and *“k”* represents the number of different ratios of emotional faces in the array. The PSE is quantified by a weighted mean, which fits a Gaussian curve. The mean represents participants’ subjective judgment of the same number of emotional and neutral faces in the array. A PSE value of 15 would indicate that there was no bias toward either emotion category (because 15 is the only number of emotional faces that “same” is the correct response to). If, however, the PSE is significantly greater than 15, this indicates that participants underestimated the number of emotional faces. In other words, people need more emotional faces in the array to judge that the number of the two categories of faces is the same.

To test whether an overestimation of emotional faces would be found in this paradigm, PSEs were submitted to a series of one-sample *t* tests, to compare the PSE to a value of 15. Analysis revealed a significant underestimation of emotional facial expressions for the angry faces, *M =* 15.14, *SD =* .51*, t*(97)* =* 2.80*, p =* .01*,* 95% CI [.04, .25], *d =* .27; and happy faces, *M =* 15.12*, SD =* .53*, t*(92)* =* 2.21*, p =* .03, 95% CI [.01, .23], *d =* .23. Independent-samples *t* test analysis revealed no significant difference between the two conditions, *t*(189) = .32, *p* = .75, *d =* .05, indicating that the underestimation of emotional faces was the same for both emotions.

**Fig. 3:**
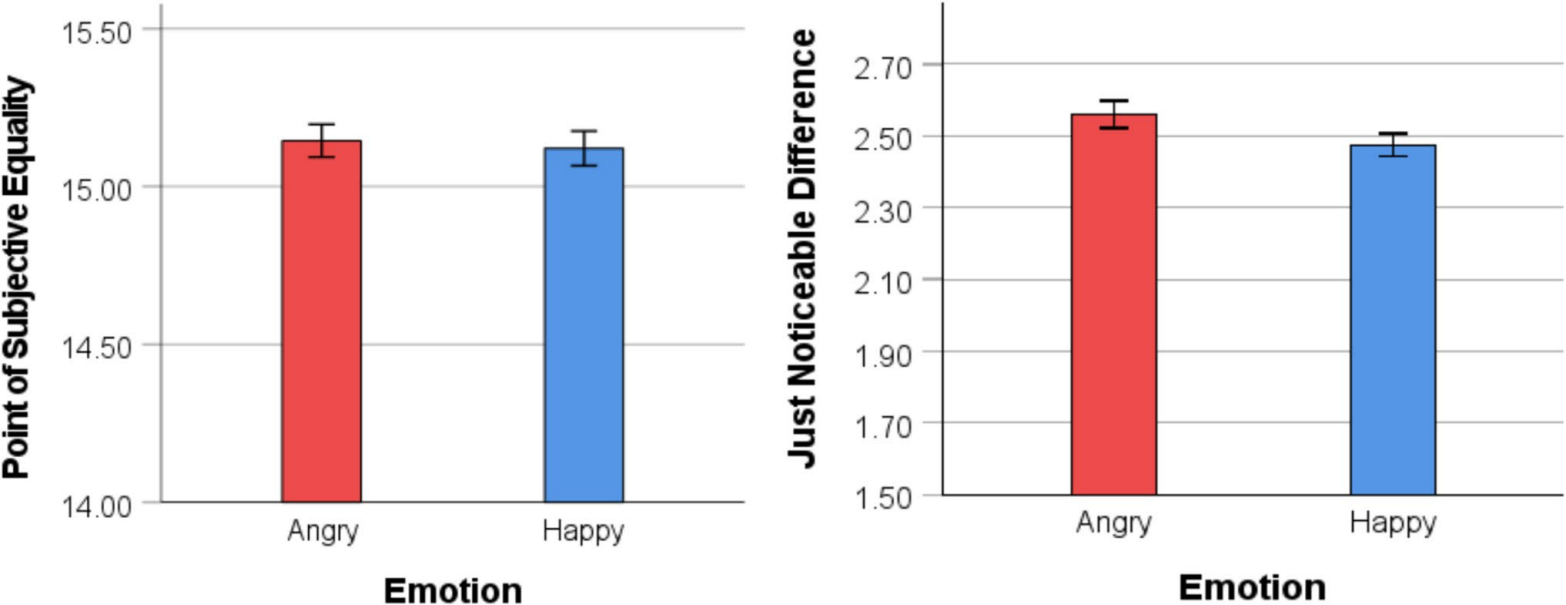
Comparison of PSE and JND for the happy and angry conditions in Experiment 1. *Note.* Mean PSE for the angry and happy conditions (left panel). A PSE greater than 15 indicates an underestimation of emotional faces. Mean JND for the angry and happy conditions (right panel). Error bars indicate one standard error

In addition to the PSE, the JND was calculated for each participant to determine the precision of judgment. The linear interpolation between the 75% threshold and the 25% threshold was applied to calculate the JND. A smaller JND indicates greater precision in judgment, while a larger JND suggests less precision in the response. An independent-samples *t* test revealed that the difference between the happy (*M* = 2.47, *SD* = .30) and angry (*M* = 2.56, *SD* = .37) conditions was not significant, *t*(189) = 1.74, *p* = .08, *d* = .25, suggesting similar difficulties when making judgments in both happy and angry conditions (see Fig. 3).

To determine whether mood states affect equivalence judgments, the scores of the anxiety and depression subscales of the DASS-21 were submitted to a series of Pearson correlations with the PSE and JND scores from the angry and happy conditions. In the angry condition, no significant associations were found between anxiety scores and PSE, *r*(98) = - .08, *p* = .45, or JND, *r*(98) = .12, *p* = .22, or depression scores and PSE, *r*(98) = - .03, *p* = .763, or JND, *r*(98) = .11, *p* = .29. Similarly, in the happy condition, no significant association was found between anxiety scores and PSE, *r*(93) = - .11, *p* = .28, or JND, *r*(93) = .07, *p* = .54, or depression scores and PSE, *r*(93) = - .06, *p* = .59, or JND, *r*(93) = .10, *p* = .35. These results demonstrate that trait mood states related to anxiety and depression do not affect performance on the emotion judgment task for either happy or angry faces.

The results of Experiment 1 revealed that participants underestimated the number of emotional faces as reflected by a PSE exceeding 15. Figure 2(A) illustrates this rightward shift, indicating that more emotional faces are needed for people to perceive them as balanced in number. This result is inconsistent with the work from Goldenberg et al. (2021), which indicated that emotional faces attract attention leading to an overestimation of the average emotion. The underestimation observed in the current study was found irrespective of emotional valence. One possible explanation for the underestimation of emotional faces is the relatively long exposure duration of 5 s used in this experiment. Studies have demonstrated that participants initially display vigilance towards briefly presented emotional faces (typically less than 500 ms), but are then avoidant of those faces when stimuli persist for longer exposure durations (i.e., 1,000 ms or more; Fox, 2002; Mogg & Bradley, 2006; Mogg et al., 2004). Therefore, an exposure duration of 5 s may have resulted in participants making their judgments after disengaging from the emotional faces and focusing on the neutral ones, leading to the underestimation of emotional faces.

The JND comparison failed to reach significance, suggesting that there is no difference in processing efficiency between happy and angry faces compared with neutral faces. In previous studies, Bucher and Voss (2019) reported that participants spent less time looking at happy faces but provided more accurate judgments than angry faces, suggesting that processing happy faces might be more effective than processing angry faces. Prolonged exposure, however, could enhance the precision of judgments for arrays with angry and neutral faces, leading to similar judgment precision in both conditions. The influence of exposure time on equivalence judgments will be explored further in Experiment 2.

## Experiment 2

Experiment 2 investigated the effect of time course on the emotional equivalence task. In Experiment 1, face arrays were presented for a relatively long exposure duration of 5 s, which may have allowed participants to shift their attention away from the emotional faces (Mogg et al., 2004). We compared 2- and 5-s exposures to examine whether exposure duration affects over/underestimation in the equivalence task. In an informal pretest, participants performed poorly when arrays of photographic faces were presented for 2 s, making it difficult to generate reliable psychometric curves for estimating the PSE. To address this issue, the stimuli were changed from photographic faces to schematic drawings. This change was expected to have the dual benefit of making the task easier while also allowing us to test whether the findings observed in Experiment 1 generalizes to schematic faces. To increase the power of this experiment, only the angry face condition was used. If exposure duration is an important factor in the underestimation of emotional faces, we expected that a 5-s exposure duration would result in an underestimation, replicating the results observed in Experiment 1, whereas a 2-s exposure duration would result in an overestimation.

### Method

#### Participants

A total of 100 participants, none of whom took part in Experiment 1, were recruited from Prolific for Experiment 2. The recruitment procedure, inclusion, and exclusion criteria were identical to those of Experiment 1. Data from two participants were excluded from the analysis because their PSEs were greater than three standard deviations from the mean, resulting in a final sample of 98 participants (40 women, *M*_age_ = 29.55 years, *SD* = 9.44).

### Stimuli

**Fig. 4:**
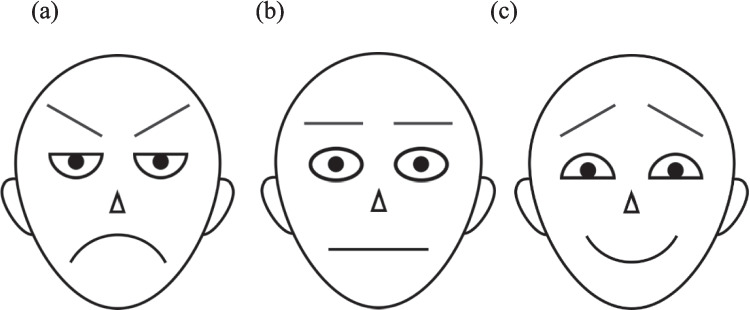
Schematic faces used to create the 6 × 5 arrangement arrays. *Note.* The angry **(A)** and neutral **(B)** faces were used in Experiments 2 and 4; the angry **(A)** and happy **(C)** faces were used in Experiment 3

The stimuli were essentially the same as those used in Experiment 1, except that the photographic faces were replaced with drawn schematic faces. Figure 4 shows the angry, neutral, and happy faces that were used to create the arrays. Each schematic face was 120 × 130 pixels in size.

#### Procedure

The experimental procedure was identical to that of Experiment 1, with the following differences. Participants completed two blocks of trials in the task. Exposure duration (2 and 5 s) was varied between blocks, and the order in which the blocks were presented was counterbalanced. Each block included 11 ratios of angry faces, and each ratio included 10 unique arrangements, totaling 110 arrays. Participants completed a total of 220 trials in this experiment.

### Results and discussion

**Fig. 5:**
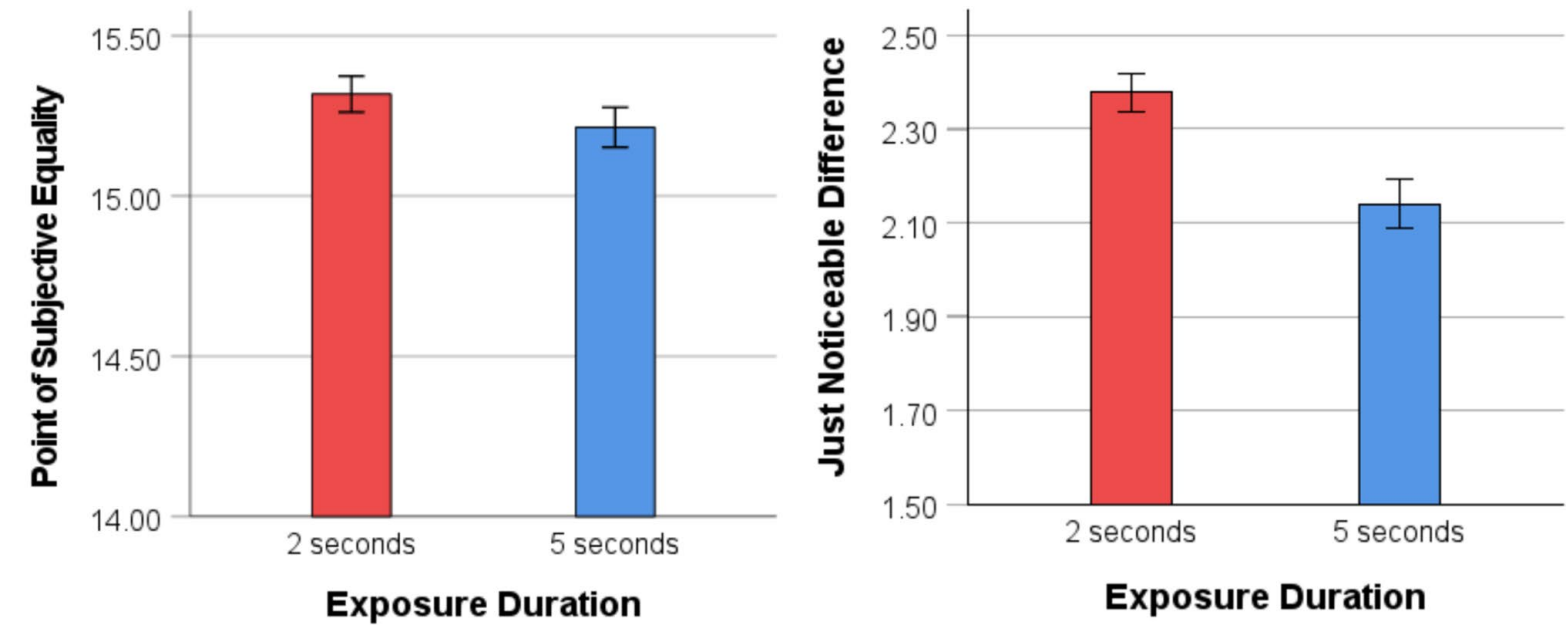
Comparison of PSE and JND for the two exposure conditions in Experiment 2. *Note.* Mean PSE for the 2- and 5-s exposure duration conditions (left panel). A PSE greater than 15 indicates an underestimation of angry faces. Mean JND for the two exposure duration conditions (right panel). Error bars indicate one standard error

The data treatment and analysis were similar to Experiment 1. A series of one-sample* t* tests comparing the PSE to a value of 15 revealed a significant underestimation of angry faces when participants viewed the arrays for both 2 s, *M =* 15.32*, SD =* .56*, t*(97*) =* 5.66*, p <* .001*,* 95% CI [.21, .43], *d =* .57, and 5 s, *M* = 15.21*, SD* = .62*, t*(97)* =* 3.44*, p* < .001*,* 95% CI [.09, .34], *d =* .35*.* A paired-samples *t* test revealed no significant difference between the two conditions, *t*(97) = 1.48, *p* = .15, 95% CI [−.04, .24], *d* = .15, indicating similar underestimation biases for both exposure durations (see Fig. 5).

A paired-samples *t* test of JND data revealed that participants were more accurate when discriminating the ratio of angry and neutral faces when exposed to arrays for 5 s (*M* = 2.14, *SD* = .53), compared with 2 s (*M* = 2.37, *SD* = .41), *t*(97) = 4.09 *p* < .001, 95% CI [.21, .62], *d* = .41 (see Fig. 5). This result aligns with the findings of Bucher and Voss (2019), who reported that longer fixation times improve the accuracy of numerosity estimation for faces. The sharper and narrower response curve for the 5-s exposure duration condition, as shown in Fig. 2(B), further demonstrates that longer exposure durations facilitate easier judgments for participants.

Pearson correlation analyses revealed that no significant association was observed between scores of mood states and judgment performance. Specifically, in the 2-s exposure, no significant correlation was found between anxiety scores and PSE, *r*(98) = −.15, *p* = .15, or JND, *r*(98) = .15, *p* = .13. There was also no significant correlation between depression scores and PSE, *r*(98) = −.10, *p* = .32, or JND, *r*(98) = .14, *p* = .16. In the 5-s exposure, anxiety scores were not significantly correlated with PSE, *r*(98) = −.11, *p* = .28, or JND, *r*(98) = −.10, *p* = .33. Similarly, depression scores were not significantly correlated with PSE, *r*(98) = −.03, *p* = .78, or JND, *r*(93) = −.11, *p* = .30. In line with Experiment 1, there was no evidence to suggest that mood states influenced judgment biases and precision in both long- and short-exposure conditions.

The results of Experiment 2 replicate the main findings of Experiment 1 using schematic faces, showing a consistent underestimation of the number of angry faces relative to neutral faces across both 2- and 5-s exposure duration conditions. These findings suggest that the tendency to underestimate the number of faces in arrays is a robust phenomenon and generalized from photographic to schematic faces. The results, however, are again inconsistent with the emotion amplification effect observed in the previous study—where longer exposures led to a greater overestimation of the angry emotion (Goldenberg et al., 2021)

One possible explanation for the underestimation is that neutral faces may not provide an appropriate baseline for comparison with angry faces. Studies have suggested that neutral faces might be perceived as more similar to angry faces under certain conditions (Lee et al., 2008), which could introduce a confound and lead to the underestimation of angry faces. Rather than comparing neutral and angry faces, Goldenberg et al. (2021) created face arrays by continuously varying the emotional intensity of faces, ranging from neutral (1 point) to extremely angry or extremely happy (50 points). In their study, each trial randomly presented an array of one to 12 faces with the average emotional intensity ranging from 10 to 40. This manipulation might enhance the contrast between emotional intensity rather than valence, hence contributing to the emotional amplification effect.

## Experiment 3

Experiment 3 explored whether the number of angry faces would be overestimated compared with happy faces. In Experiments 1 and 2, participants consistently underestimated the number of angry faces, suggesting that neutral faces may not provide a suitable contrast to angry faces because they could be categorized as broadly similar in valence (e.g., Lee et al., 2008; see also Hedger et al., 2016, for a review). To maximize the emotional contrast, we used both happy and angry faces in the same stimulus. Based on the amplification effect reported by Goldenberg et al. (2021), we hypothesized that angry faces would be overattended to compared with happy faces, leading to overestimation of their number. To test whether this effect applies consistently across different types of facial stimuli, we used both photographic and schematic faces. We expected to observe the overestimation of angry faces in both conditions, with schematic faces likely facilitating easier judgments.

### Method

#### Participants

A total of 200 participants, none of whom took part in Experiments 1 or 2, were recruited from Prolific for Experiment 3 and were randomly assigned to one of two conditions. The recruitment procedure, inclusion and exclusion criteria were identical to Experiment 1. Data from two participants in the schematic face condition and one in the photographic face condition were excluded from analysis because their PSE scores were beyond three standard deviations from the mean. The final sample included 98 participants in the schematic face condition (62 women, *M*_age_ = 31.51 years, *SD* = 9.77), and 99 participants in the photographic face condition (38 women, *M*_age_ = 31.91 years, *SD* = 10.79).

This experiment was preregistered prior to data collection (https://osf.io/ed4vy?view_only=542e469f83dd4d92a20436bbd79d094a). In the original preregistration, we planned to recruit 50 participants per condition based on an a priori power analysis, which indicated that this sample size would be sufficient to detect an effect size of *d* = .57 with 80% statistical power.^1^ However, preliminary analyses revealed trends that aligned with our hypotheses but lacked statistical significance. To improve statistical power, we increased the sample size to 100 participants per condition. This adjustment was made to enhance the reliability and generalizability of our findings, allowing for a more robust conclusion. Importantly, all data were collected and analyzed using the same experimental protocol, and no other methodological changes were introduced during the extended recruitment phase.

#### Stimuli

**Fig. 6:**
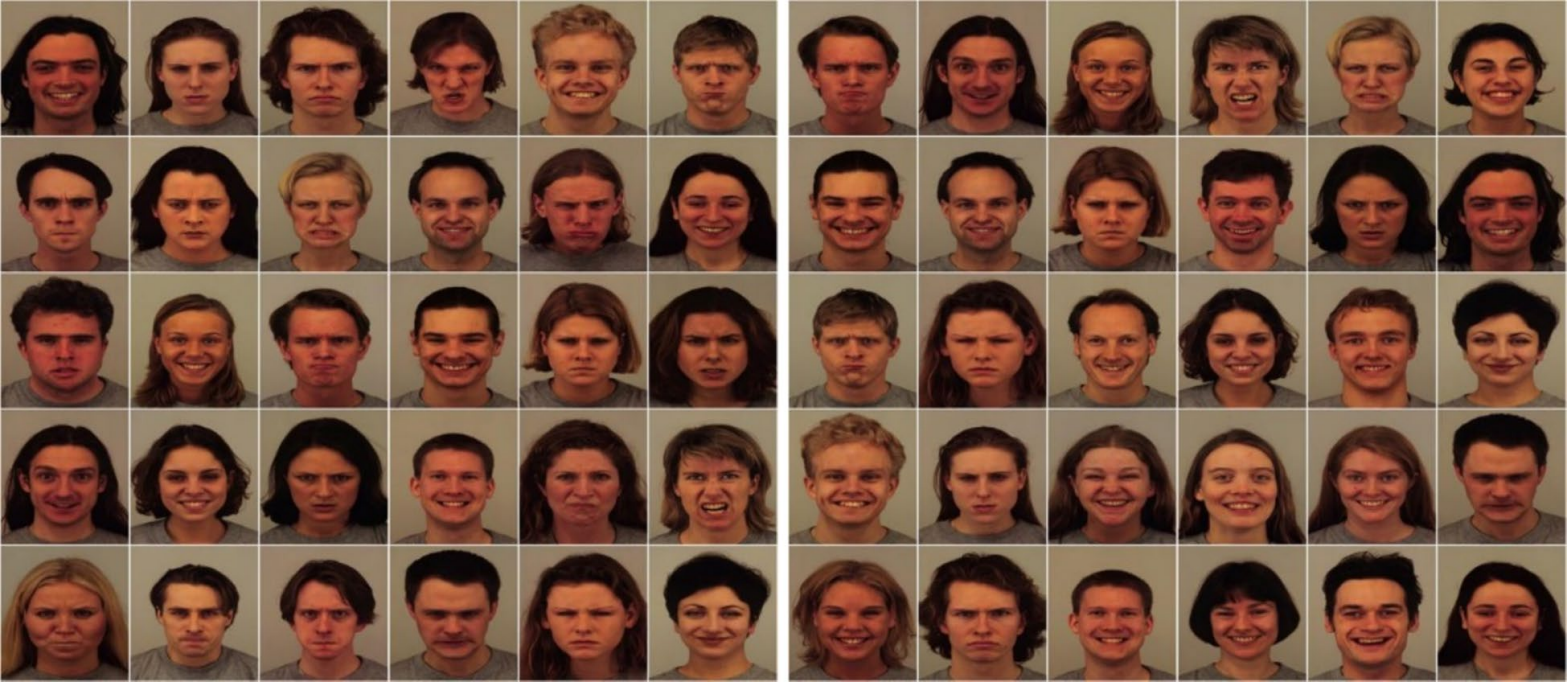
Sample photographic face arrays for Experiment 3. *Note.* Angry-dominant (i.e., 20A:10H, left panel) and happy-dominant (i.e., 20H:10A, right panel)

The stimuli were essentially the same as those used in the previous experiments, except that arrays were composed of angry and happy faces (see sample arrays of the photographic faces in Fig. 6, and the schematic faces in Fig. 4a and 4c).

#### Procedure

The experimental procedure was essentially identical to that of Experiment 1. Participants completed two blocks, each consisting of 110 trials. There were 11 different ratios of happy/angry faces ranging from 10:20 (more happy) to 20:10 (more angry).

### Results and discussion

**Fig. 7:**
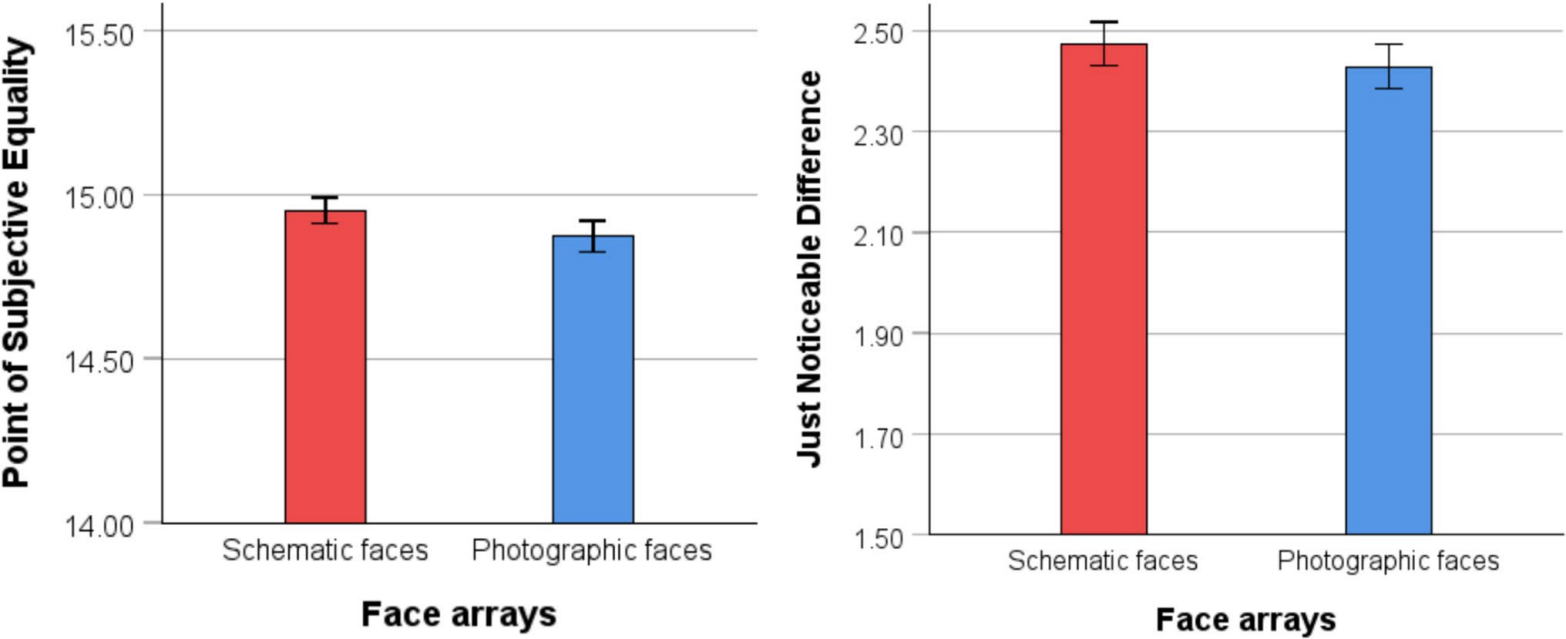
Comparison of PSE and JND for the two exposure conditions in Experiment 3. *Note.* Mean PSE for the schematic and photographic face conditions (left panel). A PSE lower than 15 indicates an overestimation of angry faces. Mean JND for the two conditions (right panel). Error bars indicate one standard error

Data treatment and analysis were consistent with those in Experiment 1. A one-sample* t* test revealed that mean PSE was significantly lower than a value of 15 for the photographic face condition (*M* = 14.88, *SD* = .44), *t*(98) = −2.62, *p* = .01, *d* = .26, indicating that participants overestimated the number of angry faces in the arrays (see Fig. 7). In other words, people need fewer angry faces in the array to judge that there is the same number of happy and angry faces. A one-sample *t* test, however, revealed no significant difference between mean PSE and a value of 15 for the schematic face condition (*M* = 14.95, *SD* = .39), *t*(97) = −1.24, *p* = .22, *d* = .13. An independent-samples *t* test also revealed no significant difference in PSE between the two conditions, *t*(195) = 1.26, *p* = .21, 95% CI [−.04, .02], *d* = .18. Analysis of JND also revealed no significant difference between the photographic face condition (*M* = 2.43, *SD* = .43) and the schematic face condition (*M* = 2.47, *SD* = .43), *t*(195) = .73, *p* = .46, 95% CI [−.08, .17], *d* = .18 (see Fig. 7).

In this experiment, we observed that participants tended to overestimate the number of angry faces. Specifically, participants needed fewer angry faces in the array compared with happy faces to perceive them as equal in number. Though, it should be noted that this effect was small and absent for schematic faces. A possible explanation is that the contrast between angry faces and happy faces is more distinct than that between angry and neutral faces (Hedger et al., 2016). Therefore, when participants had to divide their attention between angry and happy faces, they may have disproportionately focused on the angry faces. This effect was more pronounced for photographic faces because they tend to evoke stronger emotional responses (Prazak & Burgund, 2014). The findings provide the first support for the amplification effect reported by Goldenberg et al. (2021), in which angry faces were overestimated compared with happy faces.

Pearson correlation analyses revealed that, in the photographic face condition, PSE was not significantly correlated with anxiety scores, *r*(99) = .05, *p* = .64, or depression scores, *r*(99) = .04, *p* = .67. In the schematic face group, PSE was not significantly correlated with anxiety scores, *r*(98) = −.17, *p* = .10, or depression scores, *r*(98) = −.01, *p* = .94. However, a significant association was found between JND and anxiety scores: *r*(98) = .24, *p* = .02; and depression scores, *r*(98) = .21, *p* = .03, indicating that higher anxiety or depression scores were associated with poorer precision in estimation.

The correlations between mood states and JND in the schematic face condition may be attributed to the reduced emotional engagement elicited by schematic faces. Thus, participants needed to use strategies to estimate the number of faces. According to attentional control theory (Eysenck et al., 2007), anxiety and depression could impair cognitive efficiency and attentional control, thereby increasing individual differences in judgment.

## Experiment 4

Experiments 1 and 2 revealed a consistent underestimation of angry faces compared with neutral faces, which raised two potential explanations: (1) participants may have confused neutral and angry faces due to their perceived similarity in valence, or (2) attentional biases toward angry faces disrupted ensemble perception. To address these possibilities, Experiment 4 introduced a majority judgment task to reduce potential confusion and direct attention to the emotion categories. In the majority judgment task condition, participants were asked to judge whether there were more angry or neutral faces in the arrays. If the judgment bias was not due to emotional valence confusion, participants would be expected to underestimate the number of angry faces in both the equivalence and majority judgment tasks.

The response patterns in the equivalence judgment task follow a Gaussian distribution, while those in the majority judgment task conform to a cumulative Gaussian distribution. These represent different expressions of the same underlying Gaussian distribution, with the mean of each curve reflecting the central tendency of the data. In this experiment, arrays of 30 faces were presented in both the equivalence and majority judgment tasks, resulting in a response curve centered at 15, which corresponds to the actual equality point of the two emotions in the array. We compared PSE and JND to assess attentional biases and precision across the two tasks.

### Method

#### Participants

A total of 100 participants, none of whom took part in Experiments 1, 2, or 3, were recruited from Prolific for Experiment 4 and were randomly assigned to one of the two instruction conditions (*n* = 50 each). This experiment was preregistered prior to data collection (https://osf.io/tcyjz/?view_only=979d503a1b8e4d95b856d771a47cd937). The sample size was determined through an a priori power analysis using G*Power, which indicated that 50 participants were required to detect a meaningful effect size of *d* = .57 with a statistical power of 80%. After this experiment, a post hoc power analysis showed that the experiment’s statistical power was 86%. Data from one participant in the equivalence estimation condition and one participant in the majority estimation condition were excluded from the analysis because their PSE scores were three standard deviations from the mean. The final sample included 49 participants in the equivalent estimation condition (18 women, *M*_age_ = 28.38 years, *SD* = 10.24), and 49 participants in the majority estimation condition (24 women, *M*_age_ = 31.71 years, *SD* = 11.40).

#### Stimuli

The 6 × 5 arrangement arrays presented in this experiment were identical to Experiment 2.

#### Procedure

For the equivalent judgment condition, the experimental procedure was identical to that of the previous experiments: Participants judged whether the number of the two kinds of faces was the same or different. For the majority judgment condition, participants were asked to press the “A” or “N” key on the keyboard to indicate whether there were more angry faces (A) or neutral faces (N). Participants completed 220 experimental trials, consisting of two blocks of 110 trials each.

### Results and discussion

For the equivalence judgment condition, data treatment and analysis were identical to those of Experiment 1. Analysis of the PSE revealed that participants underestimated the number of angry faces in the array, *M* = 15.34, *SD* = .50, *t*(48) = 4.73, *p* < .001, 95% CI [.20, .48], *d* = .68. Pearson correlation analyses revealed no association between PSE and anxiety scores, *r*(49) = −.87,* p* = .55, or PSE and depression scores, *r*(49) = −.12, *p =* .55.

For the majority judgment condition, analyses were conducted by considering the number of “more angry” responses at each ratio of angry faces. A measure of the PSE was calculated by fitting a cumulative Gaussian function (*M* = 15, *SD* = 1) to the number of “more angry” responses across the ratios. The point of the curve at which the two responses were of equal probability was taken as the PSE. A PSE greater than 15 indicates that participants underestimated the number of angry faces in the array, consistent with the direction observed in previous experiments. A one-sample *t* test revealed that mean PSE (*M* = 15.86, *SD* = 1.07) was significantly greater than a value of 15, *t*(48) = 5.59, *p* < .001, 95% CI [.55, 1.16], *d =* .80, indicating that participants underestimated the number of angry faces. Pearson correlation analyses revealed no associations between PSE and anxiety scores, *r*(49) = .25 *p* = .08, or PSE and depression scores, *r*(49) = .22, *p =* .13.

**Fig. 8:**
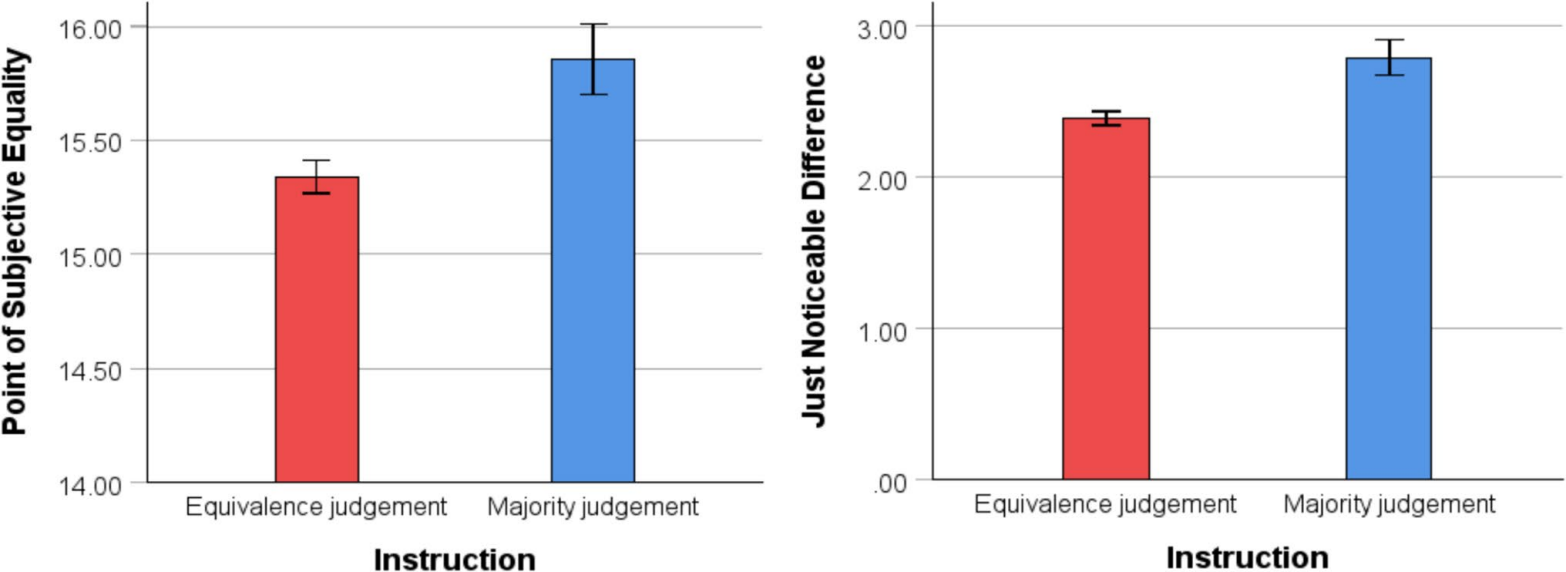
Comparison of PSE and JND for the two judgment conditions in Experiment 4. *Note.* Mean PSE for the equivalence judgment and majority judgment conditions (left panel). A PSE greater than 15 indicates an underestimation of angry faces. Mean JND for the two conditions (right panel). Error bars indicate one standard error

An independent-samples *t* test revealed that mean PSE was lower in the equivalence judgment condition compared with the majority judgment condition, *t*(68.21) = 3.05*, p* = .003*,* 95% CI [−.85, −.18], *d =* 0.62, indicating that, compared with the equivalence judgment condition, participants underestimated the number of angry faces in the array when they made majority judgments (see Fig. 8). The calculation of the JND was performed as in the previous experiments for both conditions. The comparison of the JND between both conditions revealed that the JND in the equivalence condition (*M* = 2.39, *SD* = .32) was smaller than in the majority judgment condition, (*M* = 2.79, *SD* = .82), *t*(62.41) = 3.20*, p* = .002*,* 95% CI [−.65, −.15], *d =* 0.65, indicating better precision when making equivalence judgments (see Fig. 8). Pearson correlation analyses revealed no associations between the JND and mood states in either the equivalence condition, *r*(49)* <* .25, *p* > .09, or majority condition, *r*(49) *< −*.06, *p* > .68.

This experiment demonstrated that participants consistently underestimated the number of angry faces in the arrays in both instruction conditions. These findings suggest that attentional biases, rather than perceptual confusion, underlie the judgment responses observed in the equivalence judgment task. A comparison between the two conditions revealed that the majority judgment task led to greater underestimation of angry faces than the equivalence judgment task. In line with previous research, the use of specific-emotion instructions likely reduced attention to angry faces (Lange et al., 2011; M. Williams et al., 2005), which in turn may have led to response away from angry faces (Lange et al., 2011; Yang et al., 2013).

## General discussion

The current study employed an innovative paradigm to explore how attention biases modulate emotional processing in crowds across four different experiments. Results indicated that participants consistently underestimated the number of emotional faces (angry and happy) relative to neutral faces. This underestimation effect was robust across different face types and exposure durations (Experiments 1 and 2). Contrary to the hypotheses, the results did not support the emotional amplification effect reported by Goldenberg et al. (2021). Rather than reflecting an enhanced attention to emotional faces, the current findings suggest that avoidance of emotional faces during the perception process may underlie the underestimation effect.

One possible explanation for the emotional underestimation effect is that attentional deployment may differ between extracting the mean emotion and estimating the number of faces in crowds. Extracting the average emotion primarily involves processing emotional features, and because emotional faces are typically more salient than neutral ones, people tend to automatically and preferentially attend to emotional faces. In tasks involving the estimation of the number of emotional faces, such as the paradigm used in the present study, however, participants were required not only to identify emotions but also to judge the quantity of the faces. This task requirement increased the difficulty of cognitive processing and reduced the efficiency of extracting quantity features (Chong & Treisman, 2005). Because emotional responses elicited by emotional faces can impair cognitive task performance (Blair et al., 2007; Hart et al., 2010), people might have reduced their attention to emotional faces to make sure the numerosity estimation could be completed more efficiently.

Emotion regulation may contribute to the underestimation effect by modulating attentional processes. Emotion regulation usually involves various conscious or unconscious strategies to suppress or reappraise emotional stimuli to maintain our emotional response (Gross, 2002). In the numerosity estimation task, participants may use such strategies to suppress attention toward emotional faces and reduce the potential influence of emotional response on task performance. Both angry and happy faces are typically high in arousal (Lundqvist et al., 2014) and may trigger emotional reactions that interfere with cognitive processing (Calvo & Beltrán, 2013; Hamamouche et al., 2016). To maintain emotional stability, individuals may reduce their gaze duration on angry (Fox et al., 2002) and happy faces (Mauss et al., 2007) by rapidly shifting their attention away. This strategy helps lower subjective emotional experience but also leads to shallower processing of emotional stimuli (Greif & Waring, 2018; Lench et al., 2016; Sheppes et al., 2011, 2014). This reduced processing may result in undersampling emotional faces and, in turn, underestimating their number.

Another possibility is that the high emotional arousal associated with emotional faces narrows attentional focus, making individuals less likely to sample as many emotional faces as neutral ones (Baker et al., 2013; Doi & Shinohara, 2016; Young & Cordes, 2013). However, given the relatively long exposure times used in the current study, the participants were allowed multiple attentional shifts and deeper emotional processing. Therefore, our findings suggest that the underestimation of emotional faces is more likely driven by emotional regulation mechanisms rather than by attentional narrowing alone. Future research could use methods such as EEG to explore the neural mechanisms underlying this underestimation effect. Overall, the consistent underestimation observed for angry and happy faces may be due to their high arousal levels. Such arousal could lead to an uneven allocation of attention, disrupting the well-distributed sampling for accurate ensemble perception (Haberman & Whitney, 2012).

Our findings also reveal the influence of arousal levels and resource dependence on the numerosity estimation of emotional faces. In Experiment 3, when angry and happy faces were presented simultaneously to increase the contrast of emotional intensity, participants tended to overestimate the number of angry faces. This result may be attributed to angry faces triggering stronger physiological arousal (Hedger et al., 2016) and capturing more attention than happy faces (Feldmann-Wüstefeld et al., 2011; Pool et al., 2016). Previous research has shown that when a task involves both high-arousal threat and positive stimuli, threat-related stimuli can dominate attentional resources for more extended periods (Müller-Bardorff et al., 2016; Zsidó et al., 2023), leaving fewer resources for processing positive stimuli (Pessoa, 2009). This imbalance in resource allocation may explain the overestimation of angry faces. Such patterns are consistent with the evolutionary adaptive advantage, especially for threatening stimuli, that can maintain attention capture when cognitive resources are limited (Müller-Bardorff et al., 2016).

The finding from Experiment 3 also indicates that the emotional amplification effect (Goldenberg et al., 2021) may only occur when the difference in emotional intensity reaches a certain threshold. Previous studies have shown that people can accurately estimate the average emotion of low-emotional variance crowds (Haberman & Whitney, 2009) but tend to overestimate the average emotion of high-emotional variance crowds (Goldenberg et al., 2020). These findings suggest that the processing of high-emotional variance crowds may require more complex emotion regulation mechanisms, thereby increasing the difficulty of cognitive processing (Gross, 2002) and may result in difficulty disengaging attention from emotional stimuli (Greif & Waring, 2018). In contrast, weaker emotional contrast requires fewer cognitive resources, allowing people to complete emotional regulation quickly.

The results of Experiment 4 illustrate the influence of top-down attentional control on the allocation of cognitive resources. When the task explicitly directed attention toward angry and neutral faces, top-down attentional modulation likely forced participants to allocate limited resources to neutral faces (Pessoa, 2009). To perform the estimation task effectively, they may have actively suppressed emotional responses triggered by angry faces during rapid attentional shifts. This emotional regulation strategy demands ongoing cognitive effort, which could further disrupt the integration of information from angry faces and contribute to a stronger underestimation effect (Sheppes et al., 2014). These findings indicate the role of cognitive resource availability in ensemble perception in certain situations. Understanding how the capacity of cognitive resources affects ensemble perception remains a key direction for future research.

Consistent findings across both photographic and schematic face stimuli suggest that attention is primarily guided by emotional content rather than low-level visual features. Elements like exposed teeth in photos or the curvature of the mouth in schematic faces may affect visual salience and attention prioritization (Horstmann et al., 2012; Wirth & Wentura, 2018). Previous studies have demonstrated that subtle visual differences can influence face detection. For example, angry faces were found faster in schematic stimuli (Savage et al., 2013), but results have been inconsistent for photographic stimuli (Becker et al., 2011; Savage et al., 2013). These discrepancies in visual search tasks may stem from the role of low-level features in enhancing stimulus salience, which can facilitate target detection and influence performance (Becker et al., 2011; Nummenmaa & Calvo, 2015). In contrast, ensemble perception is more robust to such visual noise (Mihalache et al., 2021; Whitney & Yamanashi Leib, 2018). Unlike visual search, which focuses on testing the ability to recognize and detect individual faces rapidly, the recognition of individual faces is not a necessary prerequisite for ensemble perception. Research has shown that people can accurately extract the average emotional tone from a crowd even when they cannot identify individual faces (Haberman & Whitney, 2009).

Furthermore, low-level visual features often act as carriers of emotional information rather than pure confound factors (Ekman & Friesen, 1978; Nummenmaa & Calvo, 2015). For example, while exposed teeth may increase salience via contrast, a bared-teeth angry expression is also perceived as more threatening, indicating that visual and emotional cues work together in emotional processing (Wirth & Wentura, 2018). The goal of ensemble perception is to summarize statistical representation for the entire group, emphasizing the dominant emotion trends within the group. Therefore, as long as sufficient emotional information is provided, low-level features may be averaged out, thereby reducing the interference in the extraction of emotional information in ensemble perception.

Given the current limited evidence, it remains unclear as to what extent low-level visual features can “be ignored” during ensemble perception, or what mechanisms underlie this process. Future research should systematically examine specific low-level features (e.g., contrast, contour clarity, or curvature) that may influence the ensemble perception independently of emotional processing. For example, future studies could present participants with facial crowds containing the same emotional categories (i.e., angry or happy expressions) but differing in low-level visual features. One group of faces might display exposed teeth, while the other group does not; alternatively, one group may have high visual contrast and the other low contrast. If participants systematically differ in their judgments of average emotion or the number of faces between these conditions, it would suggest that certain low-level features influence ensemble perception independently of emotional processing. Additionally, eye-tracking and neuroimaging techniques could explore how early visual pathways and emotion-related neural circuits, such as the dorsolateral prefrontal cortex–amygdala regulatory pathway, interact to shape ensemble perception of emotional stimuli.

This study leaves some unresolved questions that should be addressed in future research. The first limitation concerns the attention mechanism in estimating the number of emotional faces and extracting the mean emotion. In their study on low-level visual features, Lee et al. (2016) proposed that independent visual processes may be involved in perceiving both the mean value and the number of items. Although the current study suggests that estimating the number of faces may not rely on extracting the overall emotional tone, it remains unclear whether similar attention mechanisms contribute to specific stages of emotional processing in ensemble perception. Goldenberg et al. (2021) support the idea that the extraction of average emotion is derived from subset sampling. Although the results from Experiment 2 suggest that participants are unlikely to make judgments by counting the faces, otherwise, performance would have been more accurate with the 5-s exposure duration, it remains unclear whether participants aim to process as many faces as possible or if they only attend to a few faces. Future research could employ eye-tracking techniques to directly examine attention allocation.

The second limitation concerns the fact that the current study did not consider the existence of extreme values. Studies have found that people tend to both discount and overweight outliers depending on the degree of emotional variation. However, it remains unclear how strong outliers influence the underestimation effect of the number of emotional faces. Furthermore, this study did not fully investigate the impact of emotional state on emotional perception. Although anxiety and depression were assessed in each experiment, no significant association was found between these factors and the underestimation of emotional faces. This result may be because the measures reflected general emotional states in the general population, which might not significantly affect performance on the perceptual task. Previous studies have suggested that state anxiety may have a more direct regulatory effect on emotional responses in specific tasks, whereas trait anxiety is more closely related to attention bias and long-term patterns of emotional processing (Mathews & Mackintosh, 1998; Wermes et al., 2018). Future studies should distinguish between these two types of anxiety and examine how each may differently influence emotion perception.

Exploring other psychological traits closely linked to social interaction may offer a promising direction for future research on ensemble emotion perception. Traits such as alexithymia, empathy, and impulsivity could reveal additional influences on emotional processing beyond attentional bias. For example, individuals with high levels of alexithymia often struggle to identify and describe emotions (Donges & Suslow, 2017), which may result in inaccurate interpretations of both positive and negative emotional cues in a crowd. This difficulty can impair their ability to grasp the overall emotional tone of a crowd. In terms of attentional processes, future work could also examine the specific role of social anxiety. While social anxiety is known to heighten sensitivity to negative social cues, its effects on ensemble perception remain underexplored, and current evidence in this area is limited and inconsistent.

Overall, the underestimation effect observed in the current study may reflect a universal adaptive mechanism that is applicable to both negative and positive emotions, as excessive emotional arousal (regardless of positive or negative) could influence performance on cognitive tasks (Schupp et al., 2003). People may strategically suppress attention to emotional faces to optimize performance, which may explain many aspects of our behavior in social scenarios. For example, speakers try to stay calm and focus on delivering their presentation by minimizing the influence of audiences’ emotions. The findings not only strengthen our understanding of the cognitive processes underlying emotion perception and social behavior but also provide empirical evidence that could inspire interventions targeting attentional biases.

## Data Availability

All data are available via the open science framework and can be access online (https://osf.io/bv8ht/?view_only=c8060ac62e18448592c9bb66e393f12f). Materials for the study are available upon request.

## Footnotes

1 The power analysis for our sample size estimation was based on an independent-samples *t* test. This sample size also provides sufficient power for a one-sample *t* test, allowing us to detect an effect size of 0.57 with 97.5% statistical power.

## Declarations

### Open practices statements

All data are available via the Open Science Framework (https://osf.io/bv8ht/?view_only=c8060ac62e18448592c9bb66e393f12f). Experiment 1 and Experiment 2 were not preregistered. Experiment 3 and Experiment 4 were preregistered before data were collected (see https://osf.io/ed4vy/?view_only=542e469f83dd4d92a20436bbd79d094a for Experiment 3 and https://osf.io/tcyjz/?view_only=979d503a1b8e4d95b856d771a47cd937 for Experiment 4).

## References

[CR1] Ariely, D. (2001). Seeing sets: Representation by statistical properties. *Psychological Science,**12*(2), 157–162. 10.1111/1467-9280.00327 11340926 10.1111/1467-9280.00327

[CR2] Baker, J. M., Rodzon, K. S., & Jordan, K. (2013). The impact of emotion on numerosity estimation. *Frontiers in Psychology*,* 4*. 10.3389/fpsyg.2013.00521 10.3389/fpsyg.2013.00521 PMC3739062 23950754

[CR3] Beck, A. T., & Clark, D. A. (1988). Anxiety and depression: An information processing perspective. *Anxiety Research,**1*(1), 23–36. 10.1080/10615808808248218

[CR4] Becker, D. V., Anderson, U. S., Mortensen, C. R., Neufeld, S. L., & Neel, R. (2011). The face in the crowd effect unconfounded: Happy faces, not angry faces, are more efficiently detected in single- and multiple-target visual search tasks. *Journal of Experimental Psychology.,**140*(4), 637–659. 10.1037/a0024060 21744984 10.1037/a0024060

[CR5] Blair, K. S., Smith, B. W., Mitchell, D. G. V., Morton, J., Vythilingam, M., Pessoa, L., . . . Blair, R. J. R. (2007). Modulation of emotion by cognition and cognition by emotion. *NeuroImage*, *35*(1), 430–440. 10.1016/j.neuroimage.2006.11.048 10.1016/j.neuroimage.2006.11.048 PMC1862681 17239620

[CR6] Bock, R. D., & Jones, L. V. (1968). *The measurement and prediction of judgment and choice*. Holden-day.

[CR7] Bucher, A., & Voss, A. (2019). Judging the mood of the crowd: Attention is focused on happy faces. *Emotion*, *19*(6), Article 1044. 10.1037/emo0000507 10.1037/emo0000507 30265079

[CR8] Calvo, M. G., & Beltrán, D. (2013). Recognition advantage of happy faces: Tracing the neurocognitive processes. *Neuropsychologia,**51*(11), 2051–2061. 10.1016/j.neuropsychologia.2013.07.010 23880097 10.1016/j.neuropsychologia.2013.07.010

[CR9] Chong, S. C., & Treisman, A. (2003). Representation of statistical properties. *Vision Research,**43*(4), 393–404. 10.1016/S0042-6989(02)00596-5 12535996 10.1016/s0042-6989(02)00596-5

[CR10] Chong, S. C., & Treisman, A. (2005). Attentional spread in the statistical processing of visual displays. *Perception & Psychophysics,**67*(1), 1–13. 10.3758/bf03195009 15912869 10.3758/bf03195009

[CR11] Donges, U.-S., & Suslow, T. (2017). Alexithymia and automatic processing of emotional stimuli: A systematic review. *Reviews in the Neurosciences,**28*(3), 247–264. 10.1515/revneuro-2016-0049 28099136 10.1515/revneuro-2016-0049

[CR12] Doi, H., & Shinohara, K. (2016). Emotional faces influence numerosity estimation without awareness. *Cognitive Processing,**17*(4), 389–397. 10.1007/s10339-016-0774-5 27421269 10.1007/s10339-016-0774-5

[CR13] Eastwood, J. D., Smilek, D., & Merikle, P. M. (2001). Differential attentional guidance by unattended faces expressing positive and negative emotion. *Perception & Psychophysics,**63*(6), 1004–1013. 10.3758/BF03194519 11578045 10.3758/bf03194519

[CR14] El-Bar, N., Laufer, O., Yoran-Hegesh, R., & Paz, R. (2017). Over-generalization in youth with anxiety disorders. *Social Neuroscience,**12*(1), 76–85. 10.1080/17470919.2016.1167123 26988442 10.1080/17470919.2016.1167123

[CR15] Ekman, P., & Friesen, W. V. (1978). *Facial action coding system*. Springer.

[CR16] Eysenck, M. W., Derakshan, N., Santos, R., & Calvo, M. G. (2007). Anxiety and cognitive performance: Attentional control theory. *Emotion,**7*(2), 336–353. 10.1037/1528-3542.7.2.336 17516812 10.1037/1528-3542.7.2.336

[CR17] Feldmann-Wüstefeld, T., Schmidt-Daffy, M., & Schubö, A. (2011). Neural evidence for the threat detection advantage: Differential attention allocation to angry and happy faces. *Psychophysiology,**48*(5), 697–707. 10.1111/j.1469-8986.2010.01130.x 20883506 10.1111/j.1469-8986.2010.01130.x

[CR18] Fox, E. (2002). Processing emotional facial expressions: The role of anxiety and awareness. *Cognitive, Affective, & Behavioral Neuroscience,**2*(1), 52–63. 10.3758/cabn.2.1.52 10.3758/cabn.2.1.52 PMC3605771 12452584

[CR19] Fox, E., Russo, R., & Dutton, K. (2002). Attentional bias for threat: Evidence for delayed disengagement from emotional faces. *Cognition & Emotion,**16*(3), 355–379. 10.1080/02699930143000527 18273395 10.1080/02699930143000527 PMC2241753

[CR20] Frischen, A., Eastwood, J. D., & Smilek, D. (2008). Visual search for faces with emotional expressions. *Psychological Bulletin,**134*(5), 662–676. 10.1037/0033-2909.134.5.662 18729567 10.1037/0033-2909.134.5.662

[CR21] Goldenberg, A., Schöne, J., Huang, Z., Sweeny, T. D., Ong, D. C., Brady, T. F., . . . Gross, J. J. (2022). Amplification in the evaluation of multiple emotional expressions over time. *Nature Human Behaviour, 6*(10), 1408–1416. 10.1038/s41562-022-01390-y 10.1038/s41562-022-01390-y PMC10263387 35760844

[CR22] Goldenberg, A., Sweeny, T. D., Shpigel, E., & Gross, J. J. (2020). Is this my group or not? The role of ensemble coding of emotional expressions in group categorization. *Journal of Experimental Psychology: General,**149*(3), 445–460. 10.1037/xge0000651 31318257 10.1037/xge0000651

[CR23] Goldenberg, A., Weisz, E., Sweeny, T. D., Cikara, M., & Gross, J. J. (2021). The crowd-cmotion-amplification effect. *Psychological Science,**32*(3), 437–450. 10.1177/0956797620970561 33626289 10.1177/0956797620970561

[CR24] Greif, T. R., & Waring, J. D. (2018). Emotional contrast and psychological function impact response inhibition to threatening faces. *Motivation and Emotion,**42*(6), 920–930. 10.1007/s11031-018-9709-z 30581242 10.1007/s11031-018-9709-z PMC6301040

[CR25] Gross, J. J. (2002). Emotion regulation: Affective, cognitive, and social consequences. *Psychophysiology,**39*(3), 281–291. 10.1017/S0048577201393198 12212647 10.1017/s0048577201393198

[CR26] Guilford, J. P. (1954). Psychometric methods.

[CR27] Haberman, J., & Whitney, D. (2007). Rapid extraction of mean emotion and gender from sets of faces. *Current biology,**17*(17), R751–R753. 10.1016/j.cub.2007.06.039 17803921 10.1016/j.cub.2007.06.039 PMC3849410

[CR28] Haberman, J., & Whitney, D. (2009). Seeing the mean: Ensemble coding for sets of faces. *Journal of Experimental Psychology: Human Perception and Performance,**35*(3), 718–734. 10.1037/a0013899 19485687 10.1037/a0013899 PMC2696629

[CR29] Haberman, J., & Whitney, D. (2012). Ensemble Perception. In J. M. Wolfe & L. Robertson (Eds.), *From perception to consciousness* (pp. 339–349). Oxford University Press. 10.1093/acprof:osobl/9780199734337.003.0030

[CR30] Hahn, S., Carlson, C., Singer, S., & Gronlund, S. D. (2006). Aging and visual search: Automatic and controlled attentional bias to threat faces. *Acta Psychologica,**123*(3), 312–336. 10.1016/j.actpsy.2006.01.008 16524554 10.1016/j.actpsy.2006.01.008

[CR31] Hamamouche, K., Hurst, M., & Cordes, S. (2016). The effect of emotion and induced arousal on numerical processing. *Proceedings of the Annual Meeting of the Cognitive Science Society*, *38.* https://escholarship.org/uc/item/6wc1q8f2

[CR32] Hansen, C. H., & Hansen, R. D. (1988). Finding the face in the crowd: An anger superiority effect. *Journal of Personality and Social Psychology*, *54*(6), Article 917. 10.1037/0022-3514.54.6.917 10.1037//0022-3514.54.6.917 3397866

[CR33] Hart, S. J., Green, S. R., Casp, M., & Belger, A. (2010). Emotional priming effects during Stroop task performance. *NeuroImage,**49*(3), 2662–2670. 10.1016/j.neuroimage.2009.10.076 19883772 10.1016/j.neuroimage.2009.10.076 PMC2818423

[CR34] Hedger, N., Gray, K. L., Garner, M., & Adams, W. J. (2016). Are visual threats prioritized without awareness? A critical review and meta-analysis involving 3 behavioral paradigms and 2696 observers. *Psychological Bulletin,**142*(9), 934–968. 10.1037/bul0000054 27123863 10.1037/bul0000054

[CR35] Horstmann, G., & Bauland, A. (2006). Search asymmetries with real faces: Testing the anger-superiority effect. *Emotion,**6*(2), 193–207. 10.1037/1528-3542.6.2.193 16768552 10.1037/1528-3542.6.2.193

[CR36] Horstmann, G., Lipp, O. V., & Becker, S. I. (2012). Of toothy grins and angry snarls—Open mouth displays contribute to efficiency gains in search for emotional faces. *Journal of Vision,**12*(5), 7–7. 10.1167/12.5.7 22637708 10.1167/12.5.7

[CR37] Lange, W.-G., Heuer, K., Langner, O., Keijsers, G. P., Becker, E. S., & Rinck, M. (2011). Face value: Eye movements and the evaluation of facial crowds in social anxiety. *Journal of Behavior Therapy and Experimental Psychiatry,**42*(3), 355–363. 10.1016/j.jbtep.2011.02.007 21419092 10.1016/j.jbtep.2011.02.007

[CR38] Lange, W.-G., Keijsers, G., Becker, E. S., & Rinck, M. (2008). Social anxiety and evaluation of social crowds: Explicit and implicit measures. *Behaviour Research and Therapy,**46*(8), 932–943. 10.1016/j.brat.2008.04.008 18550028 10.1016/j.brat.2008.04.008

[CR39] Lee, E., Kang, J. I., Park, I. H., Kim, J.-J., & An, S. K. (2008). Is a neutral face really evaluated as being emotionally neutral? *Psychiatry Research,**157*(1), 77–85. 10.1016/j.psychres.2007.02.005 17804083 10.1016/j.psychres.2007.02.005

[CR40] Lee, H., Baek, J., & Chong, S. C. (2016). Perceived magnitude of visual displays: Area, numerosity, and mean size. *Journal of Vision,**16*(3), 12–12. 10.1167/16.3.12 26873776 10.1167/16.3.12

[CR41] Lench, H. C., Bench, S. W., & Davis, E. L. (2016). Distraction from emotional information reduces biased judgements. *Cognition and Emotion,**30*(4), 638–653. 10.1080/02699931.2015.1020767 25787937 10.1080/02699931.2015.1020767

[CR42] Lovibond, P. F., & Lovibond, S. H. (1995). The structure of negative emotional states: Comparison of the Depression Anxiety Stress Scales (DASS) with the Beck Depression and Anxiety Inventories. *Behaviour Research and Therapy,**33*(3), 335–343. 10.1016/0005-7967(94)00075-U 7726811 10.1016/0005-7967(94)00075-u

[CR43] Lundqvist, D., Flykt, A., & Öhman, A. (1998). *The Karolinska directed emotional faces*. Karolinska Institute.

[CR44] Lundqvist, D., Juth, P., & Öhman, A. (2014). Using facial emotional stimuli in visual search experiments: The arousal factor explains contradictory results. *Cognition and Emotion,**28*(6), 1012–1029. 10.1080/02699931.2013.867479 24341823 10.1080/02699931.2013.867479

[CR45] Mathews, A., & Mackintosh, B. (1998). A cognitive model of selective processing in Anxiety. *Cognitive Therapy and Research,**22*(6), 539–560. 10.1023/a:1018738019346

[CR46] Mauss, I. B., Bunge, S. A., & Gross, J. J. (2007). Automatic emotion regulation. *Social and Personality Psychology Compass,**1*(1), 146–167. 10.1111/j.1751-9004.2007.00005.x

[CR47] Mihalache, D., Lamer, S. A., Allen, J., Maher, M., & Sweeny, T. D. (2021). Anger bias in the evaluation of crowds. *Journal of Experimental Psychology: General,**150*(9), 1870–1889. 10.1037/xge0001025 33539133 10.1037/xge0001025

[CR48] Mogg, K., & Bradley, B. P. (2006). Time course of attentional bias for fear-relevant pictures in spider-fearful individuals. *Behaviour Research and Therapy,**44*(9), 1241–1250. 10.1016/j.brat.2006.05.003 16870133 10.1016/j.brat.2006.05.003

[CR49] Mogg, K., Philippot, P., & Bradley, B. P. (2004). Selective attention to angry faces in clinical social phobia. *Journal of Abnormal Psychology,**113*(1), 160–165. 10.1037/0021-843X.113.1.160 14992669 10.1037/0021-843X.113.1.160

[CR50] Müller-Bardorff, M., Schulz, C., Peterburs, J., Bruchmann, M., Mothes-Lasch, M., Miltner, W., & Straube, T. (2016). Effects of emotional intensity under perceptual load: An event-related potentials (ERPs) study. *Biological Psychology,**117*, 141–149. 10.1016/j.biopsycho.2016.03.006 26995785 10.1016/j.biopsycho.2016.03.006

[CR51] Nummenmaa, L., & Calvo, M. G. (2015). Dissociation between recognition and detection advantage for facial expressions: A meta-analysis. *Emotion,**15*(2), 243–256. 10.1037/emo0000042 25706834 10.1037/emo0000042

[CR52] Öhman, A., Lundqvist, D., & Esteves, F. (2001). The face in the crowd revisited: A threat advantage with schematic stimuli. *Journal of Personality and Social Psychology*, *80*(3), Article 381. 10.1037/0022-3514.80.3.381 10.1037/0022-3514.80.3.381 11300573

[CR53] Peirce, J., Gray, J. R., Simpson, S., MacAskill, M., Höchenberger, R., Sogo, H., . . . Lindeløv, J. K. (2019). PsychoPy2: Experiments in behavior made easy. *Behavior Research Methods*, *51*, 195–203. 10.3758/s13428-018-01193-y 10.3758/s13428-018-01193-y PMC6420413 30734206

[CR54] Pessoa, L. (2009). How do emotion and motivation direct executive control? *Trends in Cognitive Sciences,**13*(4), 160–166. 10.1016/j.tics.2009.01.006 19285913 10.1016/j.tics.2009.01.006 PMC2773442

[CR55] Pool, E., Brosch, T., Delplanque, S., & Sander, D. (2016). Attentional bias for positive emotional stimuli: A meta-analytic investigation. *Psychological Bulletin,**142*(1), 79–106. 10.1037/bul0000026 26390266 10.1037/bul0000026

[CR56] Prazak, E. R., & Burgund, E. D. (2014). Keeping it real: Recognizing expressions in real compared with schematic faces. *Visual Cognition,**22*(5), 737–750. 10.1080/13506285.2014.914991

[CR57] Purcell, D. G., Stewart, A. L., & Skov, R. B. (1996). It takes a confounded face to pop out of a crowd. *Perception,**25*(9), 1091–1108. 10.1068/p251091 8983049 10.1068/p251091

[CR58] Savage, R. A., Lipp, O. V., Craig, B. M., Becker, S. I., & Horstmann, G. (2013). In search of the emotional face: Anger versus happiness superiority in visual search. *Emotion,**13*(4), 758–768. 10.1037/a0031970 23527503 10.1037/a0031970

[CR59] Schechtman, E., Laufer, O., & Paz, R. (2010). Negative valence widens generalization of learning. *The Journal of Neuroscience,**30*(31), 10460–10464. 10.1523/jneurosci.2377-10.2010 20685988 10.1523/JNEUROSCI.2377-10.2010 PMC6634660

[CR60] Sheppes, G., Scheibe, S., Suri, G., & Gross, J. J. (2011). Emotion-regulation choice. *Psychological Science,**22*(11), 1391–1396. 10.1177/0956797611418350 21960251 10.1177/0956797611418350

[CR61] Sheppes, G., Scheibe, S., Suri, G., Radu, P., Blechert, J., & Gross, J. J. (2014). Emotion regulation choice: A conceptual framework and supporting evidence. *Journal of Experimental Psychology: General,**143*(1), 163–181. 10.1037/a0030831 23163767 10.1037/a0030831

[CR62] Schupp, H. T., Markus, J., Weike, A. I., & Hamm, A. O. (2003). Emotional facilitation of sensory processing in the visual cortex. *Psychological Science,**14*(1), 7–13. 10.1111/1467-9280.01411 12564747 10.1111/1467-9280.01411

[CR63] Stevens, S. S. (1957). On the psychophysical law. *Psychological Review,**64*(3), 153–181. 10.1037/h0046162 13441853 10.1037/h0046162

[CR64] Sussman, T. J., Jin, J., & Mohanty, A. (2016). Top-down and bottom-up factors in threat-related perception and attention in anxiety. *Biological Psychology,**121*(Pt B), 160–172. 10.1016/j.biopsycho.2016.08.006 27546616 10.1016/j.biopsycho.2016.08.006

[CR65] Ueda, Y. (2022). Understanding mood of the crowd with facial expressions: Majority judgment for evaluation of statistical summary serception. *Attention, Perception, & Psychophysics,**84*, 843–860. 10.3758/s13414-022-02449-8 10.3758/s13414-022-02449-8 PMC9001560 35292930

[CR66] Watamaniuk, S. N., & Duchon, A. (1992). The human visual system averages speed information. *Vision Research*, *32*(5), 931–941. 10.1016/0042-6989(92)90036-I 10.1016/0042-6989(92)90036-i 1604862

[CR67] Wermes, R., Lincoln, T. M., & Helbig-Lang, S. (2018). Attentional biases to threat in social anxiety disorder: time to focus our attention elsewhere? *Anxiety, Stress, & Coping,**31*(5), 555–570. 10.1080/10615806.2018.1483497 29877114 10.1080/10615806.2018.1483497

[CR68] Whitney, D., & Yamanashi Leib, A. (2018). Ensemble perception. *Annual Review of Psychology,**69*, 105–129. 10.1146/annurev-psych-010416-044232 28892638 10.1146/annurev-psych-010416-044232

[CR69] Williams, J. M. G., Watts, F. N., MacLeod, C., & Mathews, A. (1988). *Cognitive psychology and emotional disorders*. John Wiley & Sons.

[CR70] Williams, M., Moss, S., Bradshaw, J., & Mattingley, J. (2005). Look at me, I’m smiling: Visual search for threatening and nonthreatening facial expressions. *Visual Cognition,**12*(1), 29–50. 10.1080/13506280444000193

[CR71] Wirth, B. E., & Wentura, D. (2018). Furious snarling: Teeth-exposure and anxiety-related attentional bias towards angry faces. *PLOS ONE*, *13*(11). 10.1371/journal.pone.0207695 10.1371/journal.pone.0207695 PMC6258523 30481190

[CR72] Yang, J.-W., & Baek, J. (2022). Bias and sensitivity in numerosity perception of negative emotions among individuals with high social anxiety. *Scientific Reports*, *12*(1), Article 11261. 10.1038/s41598-022-15601-z 10.1038/s41598-022-15601-z PMC9252997 35788161

[CR73] Yang, J.-W., Yoon, K. L., Chong, S. C., & Oh, K. J. (2013). Accurate but pathological: Social anxiety and ensemble coding of emotion. *Cognitive Therapy and Research,**37*(3), 572–578. 10.1007/s10608-012-9500-5

[CR74] Young, L. N., & Cordes, S. (2013). Fewer things, lasting longer: The effects of emotion on quantity judgments. *Psychological Science,**24*(6), 1057–1059. 10.1177/0956797612465294 23603915 10.1177/0956797612465294

[CR75] Zsidó, A. N., Bali, C., Kocsor, F., & Hout, M. C. (2023). Task-irrelevant threatening information is harder to ignore than other valences. *Emotion,**23*(6), 1606–1617. 10.1037/emo0001189 36355669 10.1037/emo0001189

